# Oxidized Phospholipid OxPAPC Activates TRPA1 and Contributes to Chronic Inflammatory Pain in Mice

**DOI:** 10.1371/journal.pone.0165200

**Published:** 2016-11-03

**Authors:** Boyi Liu, Yan Tai, Ana I. Caceres, Satyanarayana Achanta, Shrilatha Balakrishna, Xiaomei Shao, Junfan Fang, Sven-Eric Jordt

**Affiliations:** 1 Department of Neurobiology and Acupuncture Research, The Third Clinical Medical College, Zhejiang Chinese Medical University, Hangzhou, Zhejiang Province, P.R. China; 2 Department of Laboratory and Equipment Administration, Zhejiang Chinese Medical University, Hangzhou, Zhejiang Province, P.R. China; 3 Department of Anesthesiology, Duke University School of Medicine, Durham, North Carolina, United States of America; 4 Department of Pharmacology, Yale University School of Medicine, New Haven, Connecticut, United States of America; Boston Children’s Hospital and Harvard Medical School, UNITED STATES

## Abstract

Oxidation products of the naturally occurring phospholipid 1-palmitoyl-2-arachidonoyl-*sn*-glycerol-3-phosphatidylcholine (PAPC), which are known as OxPAPC, accumulate in atherosclerotic lesions and at other sites of inflammation in conditions such as septic inflammation and acute lung injury to exert pro- or anti-inflammatory effects. It is currently unknown whether OxPAPC also contributes to inflammatory pain and peripheral neuronal excitability in these conditions. Here, we observed that OxPAPC dose-dependently and selectively activated human TRPA1 nociceptive ion channels expressed in HEK293 cells *in vitro*, without any effect on other TRP channels, including TRPV1, TRPV4 and TRPM8. OxPAPC agonist activity was dependent on essential cysteine and lysine residues within the N-terminus of the TRPA1 channel protein. OxPAPC activated calcium influx into a subset of mouse sensory neurons which were also sensitive to the TRPA1 agonist mustard oil. Neuronal OxPAPC responses were largely abolished in neurons isolated from TRPA1-deficient mice. Intraplantar injection of OxPAPC into the mouse hind paw induced acute pain and persistent mechanical hyperalgesia and this effect was attenuated by the TRPA1 inhibitor, HC-030031. More importantly, we found levels of OxPAPC to be significantly increased in inflamed tissue in a mouse model of chronic inflammatory pain, identified by the binding of an OxPAPC-specific antibody. These findings suggest that TRPA1 is a molecular target for OxPAPC and OxPAPC may contribute to chronic inflammatory pain through TRPA1 activation. Targeting against OxPAPC and TRPA1 signaling pathway may be promising in inflammatory pain treatment.

## Introduction

Transient receptor potential ankyrin 1 (TRPA1) is a non-selective cation ion channel expressed in primary sensory neurons and acts as a sensor for painful and irritating stimuli [1]. Mammalian TRPA1 is robustly activated by a wide variety of exogenous irritants that cause pain and inflammation [2]. TRPA1 is also a target of endogenous oxidative products such as 4-hydroxynonenal (4-HNE) and H_2_O_2_ that activate TRPA1 directly [3,4]. TRPA1 is a major transducing pathway by which oxidative stress produces acute nociception, allodynia, and hyperalgesia [5,6]. Activation of TRPA1 further produces neuropeptides, such as substance P (SP) and calcitonin gene-related peptide (CGRP) which mediate neurogenic inflammatory responses [2]. Pharmacological blockage or genetic ablation of TRPA1 significantly reduced allodynia and hyperalgesia in animal models of chronic inflammatory pain conditions [7–10].

1-palmitoyl-2-arachidonoyl-*sn*-glycerol-3-phosphatidylcholine (PAPC) is a naturally occurring phospholipid found in cell membranes and lipoproteins. PAPC is particularly susceptible to oxidation by endogenous oxidants produced in oxidative stress conditions [11]. Oxidation of PAPC leads to the formation of a mixture of oxidized phospholipids, ranging from epoxyisoprostanes to truncated chain derivatives that are collectively termed as OxPAPC [11]. OxPAPC was shown to accumulate in atherosclerotic lesions and at other inflamed sites in conditions such as septis and acute lung injury, where it is believed to contribute to disease progression [12]. OxPAPC levels affect a variety of signaling pathways, including kinase pathways involving protein kinase C (PKC), extracellular signal-related kinases 1 and 2 (ERK1/2) or p38 mitogen-activated protein kinase (p38-MAPK) and also increase intracellular calcium levels to promote inflammatory responses [13–15]. Several studies suggest that OxPAPC can also exert anti-inflammatory and protective effect by inhibiting the interaction of LPS with LPS-binding protein and CD14 [16–18].

The levels of reactive oxygen species contributing to the formation of OxPAPC are highly elevated in inflammatory pain conditions [2,6,19]. However, it remains unknown whether OxPAPC contributes to inflammatory pain. In the present study, we examined the effects of OxPAPC on TRPA1 expressed in a heterologous expression system and in cultured sensory neurons derived from wild-type and TRPA1-deficient mice by means of Fura-2 ratiometric [Ca^2+^]_i_-imaging. Furthermore, we examined whether OxPAPC can induce nocifensive behavior in mice *in vivo*. Lastly, we examined the presence of OxPAPC in inflamed tissue in a mouse model of chronic inflammatory pain. Our results suggest that OxPAPC is a potent endogenous agonist of TRPA1 and may contribute to chronic inflammatory pain conditions.

## Materials and Methods

### Animals

Experimental procedures were approved by the Institutional Animal Care and Use Committees of Duke University. Mice were housed at facilities accredited by the Association for Assessment and Accreditation of Laboratory Animal Care in standard environmental conditions (12-hour light–dark cycle and 23°C). Food and water were provided ad libitum. Male C57BL/6 mice (6–8 weeks old) were purchased from Jackson Laboratory (Bar Harbor, ME) and used in the present study.

### Chemicals

OxPAPC, PAPC and DMPC were purchased from Hycult Biotech (Plymouth Meeting, PA, USA); Complete Freund’s adjuvant (CFA) was purchased from Rockland Immunochemicals (Gilbertsville, PA, USA); Ionomycin was from Invitrogen (Grand Island, NY, USA). AH6809 and PF04418948 were obtained from Tocris (Minneapolis, MN, USA). Capsaicin, mustard oil and other reagents were obtained from Sigma-Aldrich (St. Louis, MO, USA)

### Cell culture and calcium imaging

Human embryonic kidney (HEK) 293 cells (ATCC, CRL-1573) were cultured in Dulbecco's modified Eagle's medium (DMEM, Lonza, Belgium) supplemented with 10% fetal bovine serum (Lonza, Belgium), 2 mM L-glutamine, 100 units/mL penicillin, and 100 μg/mL streptomycin. Cells were transfected by Lipofectamine 2000 (Invitrogen, Carlsbad, CA, USA).

Adult mouse dorsal root ganglia (DRGs) were dissociated using 0.28 Wünsch units/ml Liberase Blendzyme 1 (Roche Diagnostics, Mannheim, Germany), as described previously [20]. Neurons were cultured in Neurobasal-A medium (Invitrogen, Grand Island, NY) with B-27 supplement, 0.5 mmol/L glutamine, and 50 ng/mL nerve growth factor (Calbiochem, La Jolla, CA) on an 8-well chambered coverglass coated with poly-D-lysine (Sigma, St. Louis, MO) and mouse laminin (Invitrogen, Carlsbad, CA, USA).

For Ca^2+^-imaging of HEK293 cells, cells were used within 48 h after transfection. For DRG neurons, neurons were used 24 h after dissociation. Cells were loaded with Fura 2-AM (10 μM, Invitrogen) for 45 min in a loading buffer containing: NaCl 140, KCl 5, CaCl_2_ 2, MgCl_2_ 2, HEPES 10 (pH 7.4 adjusted with NaOH). Cells were subsequently washed three times and imaged in the loading buffer. Ratiometric Ca^2+^-imaging was performed on an Olympus IX51 microscope with a Polychrome V monochromator (Till Photonics) and a PCO Cooke Sensicam QE CCD camera and Imaging Workbench 6 imaging software (Indec). Fura-2 emission images were obtained with exposures of 0.5 ms at 340 nm and 0.3 ms at 380 nm excitation wavelengths. Ratiometric images were generated using ImageJ software. A cell was considered responsive if the peak Ca^2+^ response is above 20% of the baseline.

### Immunofluorescence

Staining was performed using 8 μm frozen sections. Sections were prefixed with acetone before staining. Primary EO6 monoclonal antibody (Avanti polar Lipids, Alabaster, AL, USA), a widely used monoclonal antibody that selectively detects oxidized phospholipids [21–23], and corresponding secondary antibodies (Invitrogen, Carlsbad, CA, USA) were used for staining. Nuclei were stained with DAPI. Images were obtained by Zeiss Imager Z1 microscope (Zeiss, Munich, Germany). For quantification of immunofluorescent staining, 3 images were randomly selected per mouse tissue. The fluorescence intensity of the stained area in each of selected images was measured by ImageJ software, and then averaged and analyzed.

### Analysis of nocifensive behavior

Mice were placed into transparent chambers and habituated for 30 minutes before testing. OxPAPC (25 μg/paw, dissolved in PBS) was injected into the hind paw of mice using 1-mL syringe and 30-gauge needle in a volume of 20 μl. Acute nocifensive behavior (licking, flinching, or biting of injected paw) was recorded with a video camera for 5 minutes and quantified thereafter.

Mechanical hyperalgesia was examined by von Frey hair analysis. Mice were habituated for 30 minutes to the wire mesh surface before testing. Paw withdrawal thresholds were determined using a series of von Frey filaments (0.008–6.00 g) pressed against the plantar surface of the hind paw in ascending order beginning with the finest fiber following standard procedures [24,25]. The minimum force (g) that caused the mouse to withdraw its hind paw away from the filament was considered as the withdrawal threshold. For each paw, a von Frey hair was applied 5 times at 10-second intervals. The threshold was determined when paw withdrawal was observed in more than 3 of 5 applications. A withdrawal response was considered valid only if the hind paw was removed completely from the platform. If the paw withdrawal response was ambiguous, the application was repeated. All behavioral tests were performed by an experimenter blinded to experimental conditions.

### Statistics

Student’s *t*-test was used for comparison of data between 2 groups. One-way or two-way ANOVA followed by Tukey post hoc test was used for comparison of ≥ 3 groups. Comparison is considered significantly different if the *p* value is less than 0.05. Data in bar graphs are expressed as means ± S.E.

## Results

### 1. OxPAPC is a selective agonist of human TRPA1 ion channels expressed in heterologous cells

**Fig 1:**
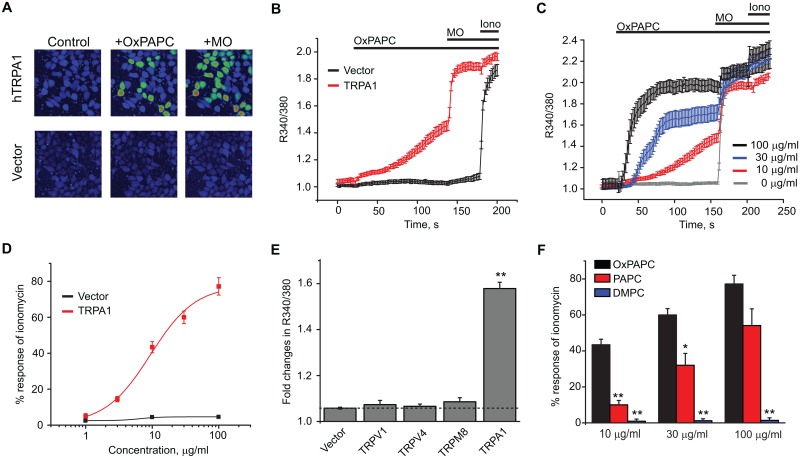
OxPAPC specifically activates human TRPA1 (hTRPA1) channels expressed in HEK293 cells. (A) Representative images derived from Fura-2 ratiometric analysis showing [Ca^2+^]_i_ changes in HEK293 cells transfected with hTRPA1 cDNA or empty vector pCDNA3.1 in response to OxPAPC (10 μg/ml) and mustard oil (MO, 70 μM). (B) Averaged Ca^2+^ responses from OxPAPC experiments in panel (A). At the end of the experiments, ionomycin (1.5 μM) was applied to activate all viable cells in the field. n > 40 cells/group. (C) Comparison of averaged Ca^2+^ responses induced by 0, 10, 30 or 100 μg/ml OxPAPC. Imaging traces are overlaid for comparison. n > 40 cells/group. (D) Dose-response analysis of OxPAPC activation of calcium influx in hTRPA1 transfected cells with vector-transfected cells as controls. The responses of OxPAPC were normalized to those of ionomycin. EC_50_ = 9.5 μg/ml. Each data point represents 5–6 separate tests. (E) Effects of OxPAPC (10 μg/ml) on hTRPA1 compared to human TRPV1, TRPV4, TRPM8 and empty vector in Ca^2+^ imaging tests. The dotted line shows the basal level obtained from cells transfected with empty vector alone. (F) Comparison of the effects of different doses of OxPAPC, PAPC and DMPC on hTRPA1 by Ca^2+^ imaging. Responses were normalized to ionomycin applied at the end of the tests. 10, 30 and 100 μg/ml of each lipid product were tested and compared. n = 6 tests/group. *p < 0.05, **p < 0.01 *vs*. OxPAPC group.

Live cell calcium imaging of human TRPA1 (hTRPA1)-expressing HEK293 cells was used to determine whether OxPAPC acts as a TRPA1 agonist. OxPAPC (10 μg/ml) robustly activated hTRPA1 expressed in HEK293 cells, while it had no detectable effect on vector (pCDNA3.1)- transfected cells (Fig 1A & 1B). Mustard oil (MO, 70 μM), a commonly used TRPA1 agonist, further activated Ca^2+^ influx into the OxPAPC-responsive cells, confirming that OxPAPC exclusively acted on TRPA1-expressing cells (Fig 1A & 1B). OxPAPC activates TRPA1 in a dose dependent manner. 10 μg/ml OxPAPC produced Ca^2+^ responses with a relatively slow onset, whereas 30 and 100 μg/ml OxPAPC produced more robust and faster activation (Fig 1C, S1 Table).

We then performed a detailed dose-response analysis of OxPAPC activation of hTRPA1. OxPAPC dose-dependently activated hTRPA1, but not empty vector-transfected HEK293 cells (Fig 1D, S1 Table). The EC_50_ of OxPAPC deduced from the curve is 9.5 μg/ml (Fig 1D). We continued to explore the selectivity of OxPAPC by comparing its effects on TRPV1, TRPV4 and TRPM8. OxPAPC (10 μg/ml) only activated TRPA1 and none of the other TRP channels (Fig 1E, S1 Table). Next, we compared the effects of OxPAPC with those of other lipid products such as PAPC and DMPC (1–2-Dimyristoyl-*sn*-glycero-3-phosphocholine) on hTRPA1 expressed in HEK293 cells. PAPC, at concentrations of 10, 30 and 100 μg/ml, also dose-dependently activated TRPA1, but with weaker potency as OxPAPC (Fig 1F). In contrast, DMPC had no effect (Fig 1F).

**Fig 2:**
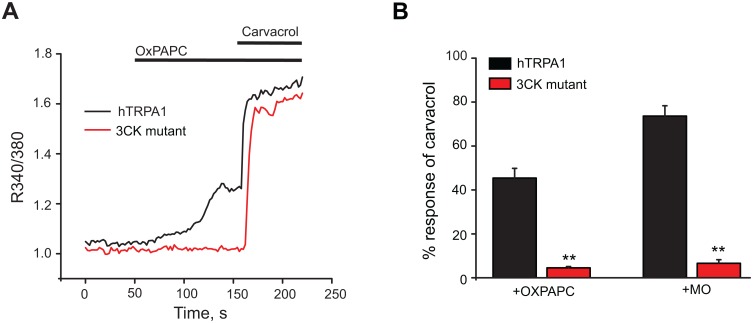
Covalent modification sites in TRPA1 are essential for OxPAPC sensitivity. (A) Representative Ca^2+^ imaging traces showing responses of hTRPA1 and hTRPA1 mutant (3CK mutant)-expressing HEK293 cells following application of OxPAPC (10 μg/ml) and carvacrol (300 μM). (B) Summary of the Ca^2+^ responses induced by OxPAPC and mustard oil (MO, 70 μM). Increase in [Ca^2+^]_i_ is displayed as percentage of [Ca^2+^]_i_ activated by a saturating dose of carvacrol (300 μM). n = 5–6 tests/group. Each test contains up to 30 cells. **p < 0.01 *vs*. hTRPA1 group.

It is well known that many reactive substances activate TRPA1 through a mechanism involving covalent modification of cysteine and lysine residues located in cytosolic N-terminus of the channel protein. We therefore examined whether these residues are needed for channel activation by OxPAPC as well. We transfected HEK293 cells with a mutant hTRPA1 channel in which critical reactive sites (C619, C639, C663, and K708, termed as TRPA1-3CK) were substituted by inert amino acid residues. These mutations have been shown to significantly reduce TRPA1 activation by many reactive chemicals [26,27]. TRPA1-3CK-transfected cells did not respond to OxPAPC and also did not respond to MO as a control reactive agonist (Fig 2A & 2B, S2 Table). TRPA1-3CK-transfected cells responded robustly to carvacrol (300 μM), a nonreactive agonist that activates TRPA1 via non-covalent mechanism (Fig 2A & 2B) [28]. These results demonstrate that OxPAPC requires the cysteine and lysine residues essential for covalent activation of TRPA1 by reactive agonists.

### 2. OxPAPC activates TRPA1 expressed in mouse dorsal root ganglion neurons

**Fig 3:**
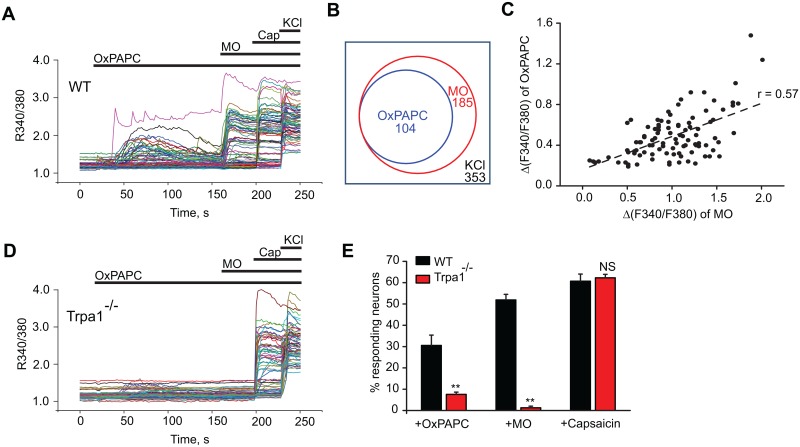
OxPAPC activates TRPA1 in cultured mouse DRG neurons. (A) Representative Ca^2+^ imaging traces showing OxPAPC responses in cultured DRG neurons isolated from WT mice. Neurons were first challenged with OxPAPC (10 μg/ml) and subsequently with mustard oil (MO, 70 μM), capsaicin (Cap, 250 nM) and KCl (40 mM). (B) Venn diagram showing the overlap of neuronal populations responding to OxPAPC, MO and Cap in neurons from WT mice. Sizes and overlap of populations are indicated by circles drawn according to scale. (C) Summary of the magnitude of OxPAPC responses *vs*. MO responses from WT mice. r = 0.57 and P < 0.00001, Pearson correlation. n = 97 neurons. (D) Representative Ca^2+^ imaging traces showing OxPAPC responses in cultured DRG neurons isolated from Trpa1^-/-^ mice. (E) Summary of the percentage of neurons responding to OxPAPC, MO and Cap isolated from WT and Trpa1^-/-^ mice. n = 5–8 tests/group, each group contains 100–300 neurons. **p < 0.01, NS: no significance (p > 0.05).

We next asked whether OxPAPC is capable of exciting primary sensory neurons and, if so, whether this occurs through activation of TRPA1. Using calcium imaging, we observed that OxPAPC (10 μg/ml) induced robust calcium influx into a subset of DRG neurons (Fig 3A). Almost all of the OxPAPC-responsive cells responded subsequently to the TRPA1 agonist, MO (Fig 3B). To determine whether OxPAPC responses were enriched in the population of neurons expressing TRPA1, we evaluated the extent to which responses to MO were correlated with responses to OxPAPC in DRG neurons. Statistical analysis of all neuronal cell responses revealed a significant positive correlation between the magnitude of the response to OxPAPC and the magnitude of the response to MO (r = 0.57 and P < 0.00001, Pearson correlation analysis, Fig 3C).

We proceeded to test if TRPA1 is required for OxPAPC-induced calcium responses in DRG neurons. DRG neurons were dissociated and cultured from TRPA1-deficient (Trpa1^-/-^) mice. We found that OxPAPC-induced calcium responses were largely abolished in Trpa1^-/-^ neurons, with only 7.6% neurons remaining responsive to OxPAPC (10 μM), as compared with 30.5% of WT neurons (Fig 3D & 3E, S3 Table). The neurons cultured from Trpa1-/- mice also lacked responses to MO, but capsaicin sensitivity was retained. (Fig 3D & 3E, S3 Table). Collectively, the above data demonstrate that TRPA1 is crucial for the response of dorsal root ganglion neurons to OxPAPC.

**Fig 4:**
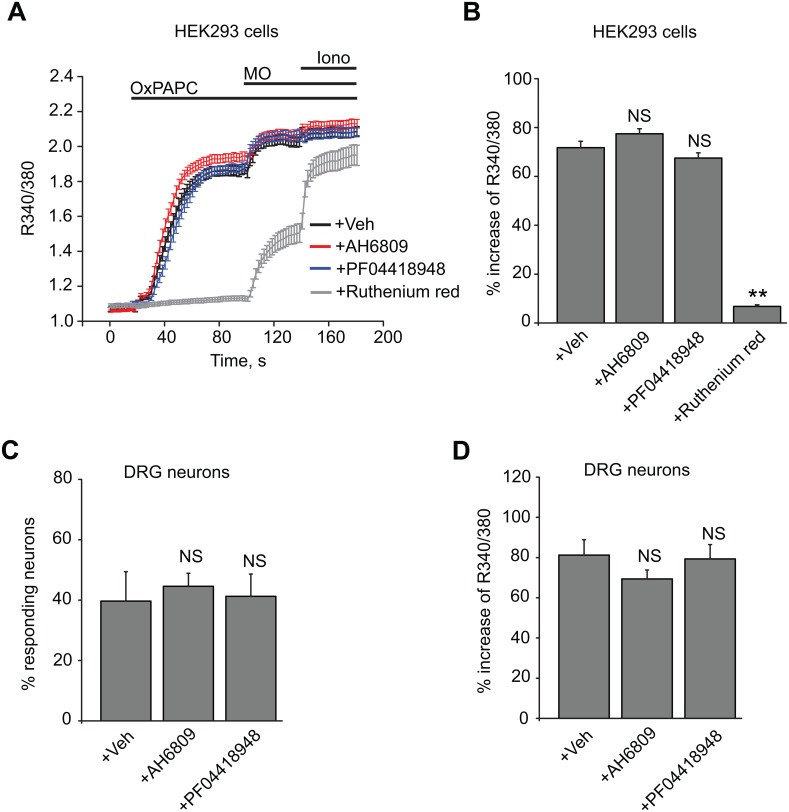
OxPAPC-induced TRPA1 activation is independent of EP2 and DP receptors in both HEK293 cells and native DRG neurons. (A) Summary of OxPAPC-induced Ca^2+^ responses in HEK293 cells expressing hTRPA1. Cells were first superfused with vehicle only (0.05% DMSO), AH6809 (10 μM), PF04418948 (20 nM) or ruthenium red (10 μM) for 5 min before recording started and then recorded in the continued presence of above treatments. Cells were challenged with OxPAPC (30 μM) and subsequently with mustard oil (MO, 70 μM) and ionomycin (1 μM). Responses of > 50 cells were averaged from each group. (B) Average peak amplitudes of OxPAPC-induced Ca^2+^ responses in HEK293 cells as shown in (A). (C) Percentages of mouse DRG neurons responding to OxPAPC in vehicle only, or in the presence of AH6809 (10 μM) or PF04418948 (20 nM). Neurons were pretreated with the antagonists for 5 min before recording and recorded in the continued presence of the treatments. Cells were challenged with OxPAPC (10 μM) for 100 s and subsequently with KCl (40 mM) for 40 s. n = 5–8 tests/group, averages from 100–200 neurons per group. (D) Maximal Fura-2 emission ratio amplitudes of OxPAPC-induced Ca^2+^ responses in mouse DRG neurons recorded in (C). **p < 0.01 *vs*. Veh, NS: no significance (p > 0.05).

It has been reported that OxPAPC can activate EP2 and DP receptors, the receptors for the prostaglandins PGE2 and PGD2 [29]. PGE2 is known to modulate TRPA1 function both *in vitro* and *in vivo* [30,31]. Therefore, it can be hypothesized that OxPAPC acts through EP2 or DP receptors to activate TRPA1 indirectly. In order to test this hypothesis, a non-selective antagonist of EP and DP receptors, AH6809, and a highly potent and selective antagonist of the EP2 receptor, PF04418948 were used to examine whether they would interfere with OxPAPC-induced TRPA1 activation [32–34]. We used HEK293 cells for these tests since these cells natively express EP2 receptors [35]. We observed that, at effective concentrations, neither AH6809 (10 μM) nor PF04418948 (20 nM) affected OxPAPC-induced TRPA1 activation in HEK293 cells (Fig 4A & 4B, S4 Table). As a positive control, the broad spectrum TRP channel blocker, ruthenium red (10 μM), robustly reduced OxPAPC-induced TRPA1 activation (Fig 4A & 4B). Next, we tested whether these two prostaglandin receptor antagonists would prevent OxPAPC-induced Ca^2+^ response in cultured mouse DRG neurons. The proportion of OxPAPC-responsive neurons (% responding neuron) and the amplitudes of OxPAPC-induced Ca^2+^ responses (% increase of R340/380) were not affected by AH6809 (10 μM) or PF04418948 (20 nM) treatment (Fig 4C & 4D, S4 Table). All together, these results suggest that OxPAPC-induced TRPA1 activation is independent of EP and DP receptors and that OxPAPC may directly activate TRPA1.

### 3. OxPAPC produces acute nocifensive behavior and persistent mechanical hyperalgesia in mice through TRPA1 activation *in vivo*

**Fig 5:**
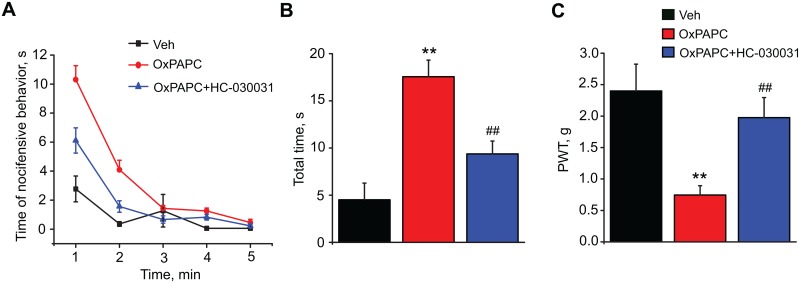
TRPA1-dependent induction of acute pain behavior and mechanical hyperalgesia by OxPAPC in mice. (A) The acute nocifensive behavior of mice quantified within the first 5 min following injection of vehicle (Veh), OxPAPC or OxPAPC+HC-030031 injection into the hind paw, plotted in 1 min intervals. (B) Cumulated nocifensive behavior response time within the total 5 min recording period of animals in experiment Fig 5 (A). (C) Paw withdraw threshold (PWT) of mice measured by von Frey hair analysis. Tests were performed 1 hour after vehicle, OxPAPC or OxPAPC+HC-030031 injection into the hind paw. n = 7–8 mice/group, **p < 0.01 *vs*. Veh, ^##^p < 0.01 *vs*. OxPAPC group.

After demonstrating that OxPAPC activated mouse DRG neurons *in vitro* we performed behavioral experiments to examine whether OxPAPC can elicit acute pain in mice *in vivo*. Intraplantar injection of OxPAPC (25 μg/paw) induced licking, biting and flinching of the injected hind paw, all indications of acute nocifensive behaviors. These behaviors occurred immediately after OxPAPC injection and gradually ceased within 5 min (Fig 5A, S5 Table). The total time the mice spent licking, biting and lifting the injected paw is summarized in Fig 5B. The acute nocifensive behaviors caused by OxPAPC were significantly attenuated by co-administration of the TRPA1 specific antagonist, HC-030031 (20 μg/paw, Fig 5A & 5B, S5 Table). Mice injected with the same amount of vehicle (PBS+1% DMSO) as a control showed minimal nocifensive responses (Fig 5A & 5B).

Next, we examined whether OxPAPC can cause persistent hyperalgesia through TRPA1 activation *in vivo*. Mice injected with OxPAPC (25 μg/paw) developed obvious mechanical hyperalgesia, measured 1 h after injection by von Frey hair analysis (Fig 5C). Co-administration of the TRPA1 inhibitor, HC-030031, significantly attenuated the mechanical hyperalgesia induced by OxPAPC (Fig 5C, S5 Table). Therefore, the above results demonstrate that OxPAPC caused both acute pain and prolonged mechanical hyperalgesia through TRPA1 activation.

### 4. Increased OxPAPC tissue levels in chronic inflammatory pain condition

**Fig 6:**
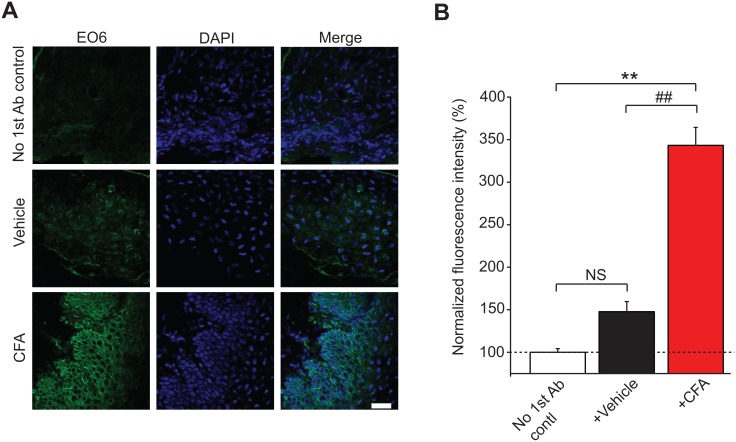
Detection of OxPAPC in the inflamed plantar skin of mice after establishment of chronic pain in the CFA model. (A) Representative immunofluorescence images of EO6 antibody staining of OxPAPC in plantar tissue of mice from vehicle (Veh)-, CFA-treated groups and no 1^st^ antibody added control (No 1^st^ Ab Contl) group. Tissues were collected 7 days after CFA or vehicle injection. Areas staining positive for EO6 antibody are shown in green. Nuclei were labeled with DAPI (blue). (B) Summary of the % increase in fluorescence of EO6 staining as shown in (A). n = 5 mice/group, **p < 0.01 *vs*. No 1^st^ Ab Contl group, ^##^p < 0.01 *vs*. Veh group, NS: no significance.

Since OxPAPC can produce acute pain and persistent mechanical hyperalgesia *in vivo*, we proceeded to ask whether tissue levels of OxPAPC were increased in animals following establishment of chronic pain. For this purpose, the mouse CFA model was used in which a chronic pain state was initiated by injection of complete Freund’s adjuvant (CFA) into the plantar surface of the hind paw. As in our previous studies, mice developed robust paw swelling and long-lasting mechanical hyperalgesia after CFA injection [24]. The inflamed plantar tissue was dissected 7 days after CFA injection and OxPAPC immunoreactivity was examined using the monoclonal antibody EO6 that specifically detects oxidized phospholipids [21–23]. We detected EO6 staining in plantar skin sections from the CFA-injected group that was significantly increased compared to staining in vehicle treated (PBS injected) mice (Fig 6A and 6B, S6 Table). Negative control experiments omitting the primary EO6 antibody (No 1^st^ antibody (Ab) control) were used as a baseline for fluorescence intensity evaluation (Fig 6A and 6B, S6 Table). Therefore, these results indicate that OxPAPC levels are significantly increased in inflamed tissues associated with chronic inflammatory pain.

## Discussion

In the present study, we have identified TRPA1 as a new molecular target for the endogenous oxidized phospholipid product OxPAPC both *in vitro* and *in vivo*. This finding is based upon the following observations: First, OxPAPC dose-dependently activated hTRPA1 expressed in HEK293 cells. The response of OxPAPC is specific since it had no effect on other TRP channels, including TRPV1, TRPV4 and TRPM8. Second, OxPAPC only activated a subset of mouse sensory neurons, which were sensitive to the TRPA1 agonist mustard oil. Third, OxPAPC-induced calcium responses in sensory neurons were largely abolished in neurons isolated from TRPA1-deficient mice. Lastly, *in vivo* studies demonstrated that injection of OxPAPC induced acute pain and persistent mechanical hyperalgesia in mice through a TRPA1-dependent mechanism.

Oxidized phospholipids exert a variety of biological effects by interacting with several cellular receptors, including scavenger receptors, platelet-activating factor receptors, peroxisome proliferator-activated receptors, prostaglandin receptors and Toll-like receptors (TLRs) [11,29]. OxPAPC can exert pro-inflammatory effects, such as enhancing inflammatory cytokine release and oxidative tissue damage, especially in vascular endothelial cells, macrophages, and smooth muscle cells [36–38]. OxPAPC also was shown to have anti-inflammatory effects in certain pathological conditions through its ability to interfere with Toll-like receptors signaling induced by microbial products which normally leads to inflammation [16,18,39,40]. Here, we provide evidence that nociceptive sensory neurons are targets of OxPAPC, with the irritant receptor TRPA1 as a molecular target, in addition to the above mentioned mechanisms.

TRPA1 is activated by oxidants and electrophiles by covalent modification of key cysteine and lysine residues within the cytosolic N terminus of the channel protein [41,42]. Transfecting HEK293 cells with a TRPA1 mutant in which these sites were converted into non-reactive residues (TRPA1-3CK) completely abolished the response to OxPAPC, while responsiveness to carvacrol, a pungent nonreactive terpene which activates TRPA1 through a non-covalent mechanism was retained. These observations suggest that OxPAPC activates TRPA1 through a covalent modification mechanism. Indeed, OxPAPC has been shown to bind to cysteine residues to regulate endothelial cell functions [43]. Thus, it is concluded that OxPAPC and other reactive TRPA1 agonists share a common mechanism activating TRPA1 through covalent modification of the same specific cysteine and lysine residues. While our pharmacological tests excluded the involvement of EP2 and DP2 prostaglandin receptors, it still remains possible that OxPAPC also modulates TRPA1 function indirectly. OxPAPC contains heterogeneous fatty acid-derived products that may act on other sensitizing receptor systems in sensory neurons. Additional pharmacological studies and patch clamp experiments will be useful to identify these pathways, validate direct effects on TRPA1 and study the potential sidedness of OxPAPC’s actions.

In the present study, we observed that PAPC, the un-oxidized precursor of OxPAPC, can activate TRPA1 as well, but to a much lesser extent when compared with OxPAPC at the same concentrations (10 or 30 μg/ml). It is possible that illumination by UV light during Ca^2+^ imaging oxidized some of the added PAPC leading to the weak TRPA1 activation we observed [44–46]. This effect can be further addressed by patch clamp recordings or the use of other (non-ratiometric) Ca^2+^-indicators excited at longer wavelengths.

Oxidative stress is a major cause of tissue damage that accompanies the development of chronic inflammation. In chronic inflammatory pain conditions, endogenous oxidants are released and may produce OxPAPC in the inflamed area. We indeed observed significant increases in immunoreactivity of OxPAPC in the inflamed tissue in a mouse model of chronic inflammatory pain. This finding demonstrates that OxPAPC is indeed generated in the inflamed tissue during chronic inflammatory pain conditions. Although the exact concentrations of OxPAPC in inflamed tissue remain unknown in inflammatory pain conditions, it is reported that in animal models of atherosclerosis the concentration of OxPAPC products in atherosclerotic lesions can reach between 100 to 200 μg/g wet tissue weight [47]. In the present study, OxPAPC began to activate hTRPA1 expressed in HEK293 cells at concentrations as low as 3.0 μg/mL and the EC_50_ is 9.5 μg/ml. If the wet tissue density is considered as 1 g/ml, then we would expect that the *in vivo* OxPAPC concentration under certain pathological conditions can significantly exceed the threshold for activation of TRPA1. Moreover, our studies showed that injection of OxPAPC produced acute pain and mechanical hyperalgesia in mice through a TRPA1-dependent mechanism. In all, our findings demonstrate that the endogenous lipid peroxidation product OxPAPC is increased in inflammatory pain conditions and may contribute to chronic inflammatory pain.

Targeting against OxPAPC may be a promising way to alleviate inflammatory pain. However, till now, there is no specific antagonist against OxPAPC production. One option is to use neutralizing antibody to block OxPAPC. It has been reported that the EO6 antibody used in the present study can abolish OxPAPC mediated inhibition of bacterial phagocytosis by macrophages *in vitro* [23]. Thus, it will be tempting to further test if EO6 can indeed neutralize the effect of OxPAPC on TRPA1 *in vitro* and see if it also works *in vivo* to blocks CFA-induced inflammatory pain.

OxPAPC has various biological effects on epithelial cells and vascular smooth muscle cells, affecting the regulation of gene expression and exerting pro- and anti-inflammatory effects [18,22,48]. Recently, functional TRPA1 expression has been reported in many non-neuronal cells such as lung fibroblast cells, epithelial and endothelial cells, smooth muscle cells and skin cells [49–51]. Activation of TRPA1 in these cells has been suggested to contribute to non-neurogenic inflammation in the airways and to melanin release from the skin [49]. Our present study has provided another possible mechanism on how OxPAPC may interact with these cells to exert biological effects. Therefore, further studies are needed to investigate to what extent the effects of OxPAPC on these cells are mediated through TRPA1 signaling.

## Conclusions

Inflammatory pain is often difficult to treat in the clinic due to insufficient understanding of the nociceptive pathways involved. Our results identify TRPA1 as a novel molecular target for oxidized phospholipid OxPAPC and suggest that endogenous OxPAPC may participate in chronic inflammatory pain through TRPA1 activation. This study further extends our current knowledge of the pathophysiological functions of OxPAPC in inflammatory pain conditions. Targeting OxPAPC and TRPA1 signaling pathway may lead to new strategies in inflammatory pain treatment.

## Supporting Information

S1 Table Raw data of OxPAPC activation of hTRPA1. (XLSX)

S2 Table Raw data of OxPAPC's effect on 3CK mutant of hTRPA1. (XLSX)

S3 Table Raw data of OxPAPC's effect on mouse DRG neurons. (XLSX)

S4 Table Raw data of the effects of EP and DP receptor antagonists on OxPAPC. (XLSX)

S5 Table Raw data of OxPAPC-induced pain in mice. (XLSX)

S6 Table Raw data of OxPAPC staining in CFA mice tissue. (XLSX)
